# The Cincinnati Enquirer

**Published:** 1889-06

**Authors:** 


The Cincinnati *Enquirer* of May 19th contains an exceedingly interesting article entitled "The Verification of Death," from the pen of Dr. William B. Clarke, of Indianapolis, Secretary of the Indiana State Homœopathic Medical Society.

Dr. Clarke thinks that "the verification of death is not carefully enough attended to by a majority of doctors." He therefore calls attention to the question of burial alive in this able paper, which was contributed to the society of which he is Secretary, and published in full in *The Enquirer*.

